# Septin 9 controls CCNB1 stabilization via APC/C^CDC20^ during meiotic metaphase I/anaphase I transition in mouse oocytes

**DOI:** 10.1111/cpr.13359

**Published:** 2022-11-10

**Authors:** Li Chen, Ying‐Chun Ouyang, Lin‐Jian Gu, Jia‐Ni Guo, Zhi‐Ming Han, Zhen‐Bo Wang, Yi Hou, Heide Schatten, Qing‐Yuan Sun

**Affiliations:** ^1^ State Key Laboratory of Stem Cell and Reproductive Biology Institute of Zoology, Chinese Academy of Sciences Beijing China; ^2^ University of Chinese Academy of Sciences Beijing China; ^3^ Department of Veterinary Pathobiology University of Missouri Columbia Missouri USA; ^4^ Fertility Preservation Lab, Guangdong‐Hong Kong Metabolism & Reproduction Joint Laboratory Reproductive Medicine Center, Guangdong Second Provincial General Hospital Guangzhou China

## Abstract

The anaphase promoting complex/cyclosome (APC/C) and its cofactors CDH1 and CDC20 regulate the accumulation/degradation of CCNB1 during mouse oocyte meiotic maturation. Generally, the CCNB1 degradation mediated by APC/C^CDC20^ activity is essential for the transition from metaphase to anaphase. Here, by using siRNA and mRNA microinjection, as well as time‐lapse live imaging, we showed that Septin 9, which mediates the binding of septins to microtubules, is critical for oocyte meiotic cell cycle progression. The oocytes were arrested at the MI stage and the connection between chromosome kinetochores and spindle microtubules was disrupted after Septin 9 depletion. As it is well known that spindle assembly checkpoint (SAC) is an important regulator of the MI‐AI transition, we thus detected the SAC activity and the expression of CDC20 and CCNB1 which were the downstream proteins of SAC during this critical period. The signals of Mad1 and BubR1 still remained on the kinetochores of chromosomes in Septin 9 siRNA oocytes at 9.5 h of in vitro culture when most control oocytes entered anaphase I. The expression of CCNB1 did not decrease and the expression of CDC20 did not increase at 9.5 h in Septin 9 siRNA oocytes. Microinjection of mRNA encoding Septin 9 or CDC20 could partially rescue MI arrest caused by Septin 9 siRNA. These results suggest that Septin 9 is required for meiotic MI‐AI transition by regulating the kinetochore‐microtubule connection and SAC protein localization on kinetochores, whose effects are transmitted to APC/C^CDC20^ activity and CCNB1 degradation in mouse oocytes.

## Graphical abstract

Mechanism of Septin 9 depletion‐caused metaphase I (MI)/anaphase I (AI) transition failure during meiotic maturation in mouse oocytes. Septin 9 may play an important role in regulating the MI/AI transition by influencing the stability of kinetochore‐microtubule connections in mouse oocytes. In wild types, Septin 9 allows CCNB1 degradation, which in turn causes MI‐to‐AI transition and the first polar body extrusion. Conversely, depletion of Septin 9 disrupts CCNB1 degradation by sustaining spindle assembly checkpoint (SAC) activation and downregulating APC/C^CDC20^ activity. Sustained SAC activation is caused by unstable connections between kinetochores and microtubules in Septin 9‐depleted oocytes. Accordingly, Septin 9‐depleted oocytes arrested at MI stage and did not extrude the first polar body.

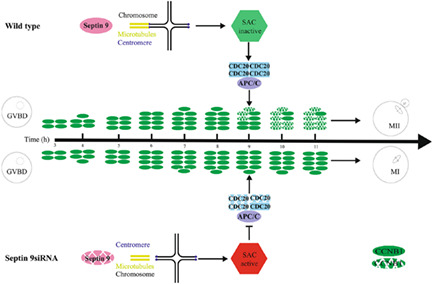

## 1 INTRODUCTION

The most important event for producing healthy offspring is proper separation of chromosomes and their equal distribution to daughter cells in every cell cycle of both germ cells and somatic cells. In order to avoid aneuploidy, the oocytes have developed a highly conserved mechanisms to ensure that homologous chromosomes and sister chromosomes separate correctly during oocyte meiosis.^1^, ^2^ The stable connection of kinetochores and microtubules is necessary for accurate separation of chromosomes in both mitosis and meiosis. Kinetochores play important functions to sense microtubule tension imbalance or microtubule‐chromosome connection loss by the spindle assembly checkpoint (SAC).^3^, ^4^, ^5^, ^6^ SAC is a highly conserved supervision mechanism that can delay the onset of anaphase until all chromosomes are properly attached to the spindles and the chromosomes are properly aligned on the equatorial plates.^7^, ^8^, ^9^ The main components of SAC include mitotic arrest‐deficient (Mad)1–3, budding uninhibited by benzimidazole (Bub)1–3, BubR1, Aurora B and Mps 1.^10^, ^11^, ^12^, ^13^, ^14^, ^15^, ^16^ Mad1 and Mad2 are the last two checkpoint proteins that attach to the kinetochore, and in addition, BubR1 is required for Mad1 and Mad2 loading at the kinetochore.^17^, ^18^, ^19^ Studies have shown that Mad2 not only can directly sense the connection between microtubules and kinetochores, but also inhibit CDC20 function.^20^ Studies have also shown that CDC20 is the downstream protein of SAC.^21^ CDC20 is the activator of APC/C, a complex composed of 11 subunits with the activity of ubiquitin ligase, which can promote the metaphase‐to‐anaphase transition.^8^ APC/C has ubiquitin activity only when it binds to CDC20 or CDH1.^22^ The SAC pathway, which ultimately transmits suppressed signals to APC/C^CDC20^ through Mad1 and Mad2, prevents the metaphase‐to‐anaphase transition until all chromosomes are properly connected to the bipolar spindle.^23^ When chromosomes are properly connected, SAC is inactivated, and the ubiquitase activity of APC/C^CDC20^ leads to the degradation of CCNB1 and then promotes the metaphase‐to‐anaphase transition.

Generally, the CCNB1 degradation caused by APC/C^CDC20^ is essential for the transition from metaphase to anaphase. CCNB1 is a regulatory subunit of MPF, which is highly conserved and was first found in frog eggs, and named for its ability to promote oocyte maturation.^24^ CCNB1 plays a key regulatory role in the meiosis of oocytes, and its regular synthesis and degradation promotes cell cycle progression. The synthesis and accumulation of CCNB1 is necessary for first meiosis resumption, and its continuous accumulation of CCNB1 further increases the activity of CDK1 and promotes the entry into meiotic metaphase.^25^ Degradation of CCNB1 is necessary for the metaphase I (MI)‐to‐anaphase I (AI) transition in the first meiosis of oocytes. APC/C^CDC20^ plays a key role in degrading CCNB1, while the activity of APC is regulated by the phosphorylation state of CDK1.^26^ Therefore, the activation of CDK1 caused by CCNB1 accumulation also in turn triggers CCNB1 degradation at the MI‐to‐AI transition during first meiosis. The degradation of CCNB1 turns off the activity of CDK1 and promotes the extrusion of the first polar body.

Septins comprise a conservative family of GTP‐binding proteins and they are widely expressed in eukaryotes but not in plants.^27^ Ford and Pringle first reported the possibility of Septins interacting with microtubules in yeast.^28^ Its members are divided into four groups: Septin 2 group, Septin 3 group, Septin 6 group and Septin 7 group. *Sept 9* is a member of the *Sept* gene family and it belongs to the *Sept 3* group, being involved in cell division.^29^ *Sept 9* gene is located on chromosome 17Q 25.3, a segment of the chromosome that is a common loss of heterozygosity in sporadic ovarian and breast cancers. Studies have confirmed that deletion of the *Sept 9* gene affects cytoplasmic division, spindle assembly and the production of polyploidy or aneuploidy in heterogeneous cells, which interferes with cell stability.^30^, ^31^, ^32^ The *Sept 9* gene is related to a variety of human diseases and plays a role in the development and progression of tumours.^33^, ^34^, ^35^, ^36^ Septin 9 recruits other proteins to specific sites in the cytoplasm through the interaction between nucleotides, tubulin and actin by biochemical structural analysis. The silencing of *Sept 9* gene results in abnormal cell division and karyotypic cells.^37^ Some studies proved that the inhibitory effect of *Sept 9* gene on apoptosis may promote the progression of breast cancer.^38^ Recent studies have supported *Sept 9* as a potential proto‐oncogene.^33^, ^34^ Studies also have shown that when the *Sept 9* gene is knocked out in active cells, the cells cannot undergo cytoplasmic division and become multinucleated, probably caused by the dysfunction of related functions of spindles, the disordered separation of chromosomes, and the instability of the cell genome.^30^

In this study, we investigated the functions of Septin 9 during oocyte meiosis and found that Septin 9 plays a key role in regulating the meiotic metaphase‐to‐anaphase transition and meiotic maturation of oocytes probably by indirectly influencing the stability of CCNB1 stability through the SAC‐APC/C^CDC20^ cascade in mouse oocytes.

## 2 MATERIALS AND METHODS

### 2.1 Antibodies and reagents

Antibodies were as follows: Anti‐Septin 9 is a rabbit polyclonal antibody (Abclonal Technology, Cat# A8657, RRID: AB_2772183); Anti‐β‐actin is a rabbit monoclonal antibody (Abclonal Technology, Cat# AC026, RRID: AB_2768234); Anti‐α‐tubulin‐FITC is a mouse monoclonal antibody (Sigma Aldrich, Cat# F2168, RRID: AB_476967). Anti‐CCNB1 is a mouse monoclonal antibody (Abcam, Cat# ab72, RRID: AB_305751); Anti‐CDC20 is a rabbit polyclonal antibody (Abclonal Technology, Cat# A15656, RRID: AB_2763063). Monoclonal Anti‐Myc antibody is produced in the mouse (Sigma Aldrich, Cat#M4439, RRID: AB_439694); Anti‐MAD1 is a rabbit polyclonal antibody (GeneTex, Cat# GTX105079, RRID: AB_11173437); Anti‐BubR1 is a sheep polyclonal antibody (Abcam, Cat# 28193, RRID: AB_725786). Anti‐centromere antibody (ACA) is a human polyclonal antibody (Antibodies Incorporated, 1:50, Cat# 15‐234‐0001, RRID: AB_2687472); Cy5‐AffiniPure Donkey Anti‐Human IgG (H + L) antibody (Jackson ImmunoResearch Labs Cat# 709‐175‐149, RRID:AB_2340539). Alexa Fluor @488‐conjugate Goat anti‐Rabbit Immunoglobulin G (IgG; H + L) and Alexa Fluor @594‐conjugate Goat anti‐Rabbit IgG (H + L) (Thermo Fisher Scientific, Catalog# A‐11008, RRID: AB_143165, Catalog# A‐11012, RRID: AB_141359); TRITC‐conjugated goat anti‐mouse IgG (H + L) (Jackson ImmunoResearch Laboratories, Inc, and sub‐packaged by Zhongshan Golden Bridge Biotechnology Co. Ltd. Cat#Zf‐0313, RRID: AB_2571577). With the exception of specific mention, other reagents were purchased from Sigma‐Aldrich.

### 2.2 Mice and ethics statement

Six‐to‐eight‐week‐old female ICR mice were purchased from SPF (Beijing) Biotechnology Co, Ltd. All experimental protocols and animal handling procedures were conducted in conformity to the standard of the Animal Research Committee of the Institute of Zoology (IOZ) at the Chinese Academy of Sciences. Under the standard experimental operation, the mice were killed by cervical dislocation, and oocytes were collected from their ovaries.

### 2.3 Oocyte collection and culture

We collected the GV stage oocytes from the ovary and the surrounding cumulus cells were removed mechanically with a pipette. Next these oocytes were incubated in M2 medium with or without 200 μM IBMX under mineral oil, and then the medium was placed in a constant temperature incubator at 37°C, 5% CO_2_. The purpose of IBMX use was to maintain oocytes at the GV stage. At last, oocytes were collected for different experiments.

### 2.4 Real‐time polymerase chain reaction

Total RNA was extracted from 80 oocytes using the RN‐easy micro purification kit (Qiagen) following the instructions. The first‐strand complementary DNA (cDNA) was generated using oligo (dT) primers, and then a list of the primers of Septin 9 fragment was made as follows: forward: 5′‐CAGGAGTCACACGGACCTC‐3′. Reverse: 5′‐CGGGCTCTGAGTTCTTCACC‐3′. Gapdh as a reference gene, generally, the primers were: 5′‐CCCCAATGTGTCCGTCGTG‐3′; Reverse: 5′‐TGCCTGCTTCACCACCTTCT‐3′. SYBR Premix (Kangwei) was used in Roche Light Cycler 480 and the mRNA levels of Septin 9 and Gapdh were detected by real‐time quantitative PCR (QPCR) analysis (Roche 480; Roche Diagnostics).

### 2.5 Immunofluorescence and confocal microscopy

4% paraformaldehyde was used to fix the oocytes at room temperature (RT) for 30 min, and then 0.5% Triton X‐100 was used to permeabilize oocytes for 20 min at RT. Next, we blocked oocytes in 1% bovine serum albumin (BSA) for 1 h at RT and then incubated over night at 4°C with anti‐Septin 9 Rabbit pAb (1:50), Anti‐α‐tubulin‐FITC antibody (1:100). Next, we washed the oocytes three times with washing buffer (0.1% Tween 20% and 0.01% Triton X‐100 in PBS) and then labelled them with F488‐conjugated goat anti‐rabbit IgG (1:100), F594‐conjugated goat anti‐rabbit IgG (1:100) for 2 h at RT. Finally, DAPI was used to stain DNA for 15 min and oocytes were mounted on glass slides with antifade mounting medium (DABCO). At last, oocytes were visualized with a Carl Zeiss LSM 780 confocal microscope.

### 2.6 Chromosome spreads

First, oocytes were treated with Acid Tyrode's solution (Sigma‐Aldrich) to remove the zona pellucida properly for 2 min at RT. Then, we transferred the oocytes into pre‐warmed M2 medium for a short recovery, and subsequently, the oocytes were transferred onto a clean glass slide and exposed to a solution of 1% paraformaldehyde (PFA) in distilled H_2_O (pH 9.2) containing 0.15% Triton X‐100 and 3 mM dithiothreitol as previously reported.^39^ The slides were placed in a half‐open humidified chamber to dry slowly for 2 h. After three washes (5 min each wash) with washing buffer (PBS containing 0.1% Tween‐20 and 0.01% TritonX‐100), the fixed oocytes were blocked with 2% BSA in PBS for 1 h at RT or overnight at 4°C. The oocytes were then incubated with primary antibodies overnight at 4°C. After three washes (10 min each wash) with washing buffer (PBS containing 0.1% Tween‐20 and 0.01% Triton X‐100), the slides were then incubated with corresponding secondary antibodies for 2 h at RT. Finally, DAPI was used to stain DNA for 15 min and oocytes were mounted on glass slides with antifade mounting medium (DABCO). At last, oocytes were visualized with a Carl Zeiss LSM 780 confocal microscope.

### 2.7 Immunoblotting analysis

Samples (each containing 150 oocytes) were mixed with 2 × SDS loading buffer and boiled for 5 min in a boiling water bath for the next steps. Target proteins were separated and then transferred to polyvinylidene fluoride membranes. And then 5% BSA with TBST was used to block target proteins for 1 h at RT. The membranes were incubated over‐night at 4°C with Septin 9 Rabbit pAb (1:1000), mouse monoclonal anti‐CCNB1 (1:500) antibody, rabbit polyclonal anti‐CDC20 antibody (1:1000), or mouse monoclonal anti‐β‐actin antibody (1:2000). TBST again was used to wash oocytes for three times and then target proteins were incubated with specific secondary antibodies (1:3000), respectively, for 1 h at RT. Finally, the protein bands were detected using Thermo Supersignal West Pico chemiluminescent substrate (Thermo Fisher Scientific).

### 2.8 Microinjection of mRNA and siRNA

Nikon Diaphot ECLIPSE TE 300 (Nikon UK Ltd) was used to perform microinjections of oocytes within 30 min. A volume of 20 μM *Sept 9* siRNAs or control siRNA from JTSBIO Co., Ltd was used for microinjection into the cytoplasm of oocytes to deplete Septin 9 and as a control. A volume of 20 μM *Cdc20* siRNA from JTSBIO Co., Ltd was microinjected into the cytoplasm of oocytes to deplete CDC20. The same amount of control siRNA was injected as a control, as described in a previous report.^40^ After injection, the GV oocytes were arrested at the GV stage in M2 medium with 200 μM IBMX for 24 h to allow depletion of Septin 9 or CDC20. Next, we fully washed the oocytes and transferred them into IBMX‐free medium. Next, to examine the expression level of Septin 9‐mCherry, Myc‐CDC20 or CCNB1‐GFP dynamics and trace the temporal and spatial extrusion of the first polar body (PBE), 20 ng/μl *Sept 9* mRNA or *Cdc20* mRNA, CCNB1 mRNA (40 ng/μl); MAP4‐GFP mRNA (200 ng/μl) and H2B‐mcherry mRNA (50 ng/μl) was injected into the GV oocytes. Each oocyte was microinjected with approximately 10 pl of *Sept 9* siRNA, *Cdc20* siRNA, *Sept 9* mRNA, *Cdc20* mRNA or control siRNA or MAP4‐GFP mRNA or H2B‐mcherry mRNA and control mRNA. Each experiment was performed three times separately and no less than 150 oocytes were used in each group.

### 2.9 Time‐lapse live imaging experiments

CCNB1‐GFP, MAP4‐GFP, H2B‐mcherry dynamics was filmed on a Perkin Elmer precisely Ultra VIEW VOX Confocal Imaging System equipped with an incubator chamber (at 37°C, 5% CO_2_) filled with M2 medium covered with a layer of paraffin oil. The image was obtained by the Volocity 6.0 software. Septin 9 siRNA‐injected oocytes and control siRNA‐injected oocytes were incubated in M2 medium within 200 μM IBMX for 24 h (at 37°C, 5% CO_2_). And next we released the oocytes from M2 medium and prepared them for time‐lapse imaging. Before designing this experiment, we had set up a procedure with shooting every 30 min to track and record the expression changes for CCNB1‐GFP for 14 h and MAP4‐GFP, H2B‐mcherry for 14 h.

### 2.10 Statistical analysis

All experiments were performed at least three times. Data are shown as mean ± standard error and the number of oocytes marked is shown as (*n* = ). Statistical analyses were processed by Student's *t* test using Prism 5 (GraphPad Software), with *p < 0.05 regarded as significant. ImageJ software (National Institutes of Health) and Photoshop CS5 (Adobe) were used to analyse the images. At last all images were composed by Illustrator CC5 (Adobe).

## 3 RESULTS

### 3.1 Expression and subcellular localization of Septin 9 during oocyte meiotic maturation

**FIGURE 1:**
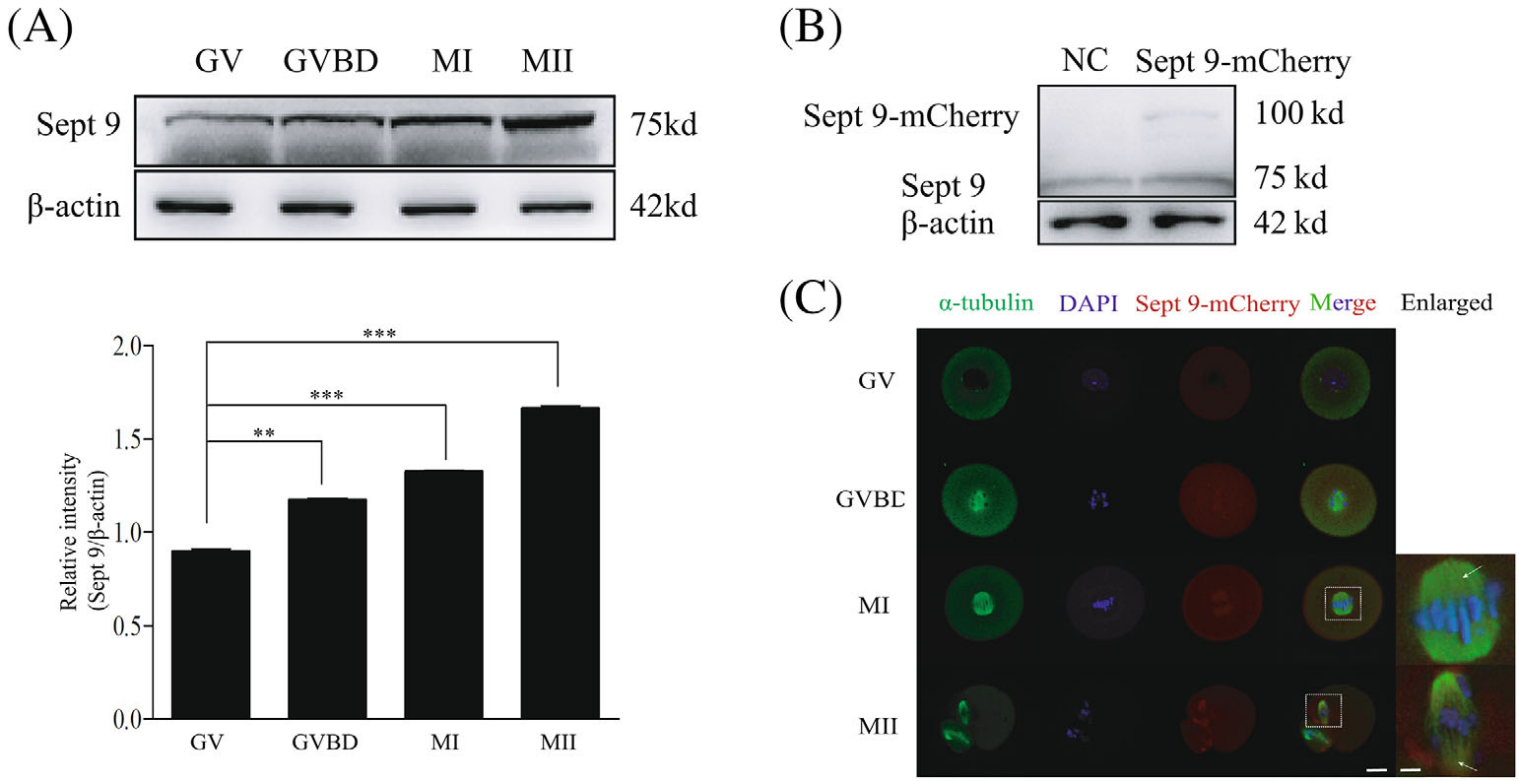
Expression and subcellular localization of Septin 9 during meiotic maturation in mouse oocytes. (A) Expression level of Septin 9 was detected by Western blotting. Samples were collected after 0, 2, 8 or 14 h of culture, corresponding to the GV, GVBD, MI and MII stages, respectively. The molecular weights of Septin 9 and β‐actin were about 75 and 42 Kd, respectively. Each sample contained 150 oocytes. The relative intensity of Septin 9 was analysed by grayscale analysis using the software Image J. Levels of expression were normalized to the levels of β‐actin. Error bars are mean ± SEM. (B) Western blotting results for Septin 9, Septin 9—mCherry and β‐actin in the mCherry—Sept 9 mRNA injected oocytes and control mRNA injected oocytes. The molecular weights of Septin 9—mCherry was about 100 Kd. Each sample contained 150 oocytes. (C) Subcellular localization of Septin 9 as revealed by immunofluorescent staining. Oocytes at the GV, GVBD, MI and MII stages were stained with antibody against α‐tubulin to visualize spindle (Green) and were stained with DAPI to visualize DNA (Blue). Each sample contained 50 oocytes. Scale bars: 20 μm. Enlarged panels show high magnification views of the boxed areas. Arrows indicate the co‐localization of Septin 9 and spindle. Scale bar: 1 μm (enlarged panels). GV, germinal vesicle; GVBD, germinal vesicle breakdown; MI, metaphase I; MII, metaphase II; SEM, standard error of mean. Data are mean ± sem. **p < 0.01; ***p < 0.001. All of the experiments were repeated at least three times, and representative results are shown.

We collected oocytes at the GV, GVBD, MI and MII stages to test the expression level and subcellular localization of Septin 9 during oocyte meiotic maturation. As shown in Figure 1A, Western blotting results showed that Septin 9 was expressed at all stages. Due to the fact that the anti‐Septin 9 antibody cannot be used for immunofluorescent staining, we constructed *Sept 9‐mCherry* plasmid and then injected its mRNA into the oocytes to observe the subcellular localization of Septin 9. As shown in Figure 1B, we verified that the *Sept 9‐mCherry* plasmid was successfully constructed and its coding protein was successfully expressed. To check the subcellular localization of Septin 9 during meiotic maturation, oocytes were injected with *mCherry ‐Sept 9* mRNA and collected at different stages for immunofluorescent staining. As shown in Figure 1C, Septin 9 was observed to distribute in the cytoplasm at GV to GVBD stages. Shortly after GVBD (1–2 h of culture), Septin 9 began to migrate to the periphery of chromosomes until the MI spindle was formed. At MI and MII stages, Septin 9 co‐localized to the periphery of the spindle, which is consistent with the report on the interaction of endogenous Septin 9 with microtubules and F‐actin.^41^ These results suggested that Septin 9 might function in mouse oocyte meiotic maturation.

### 3.2 Depletion of Septin 9 affects the PBE rather than GVBD

**FIGURE 2:**
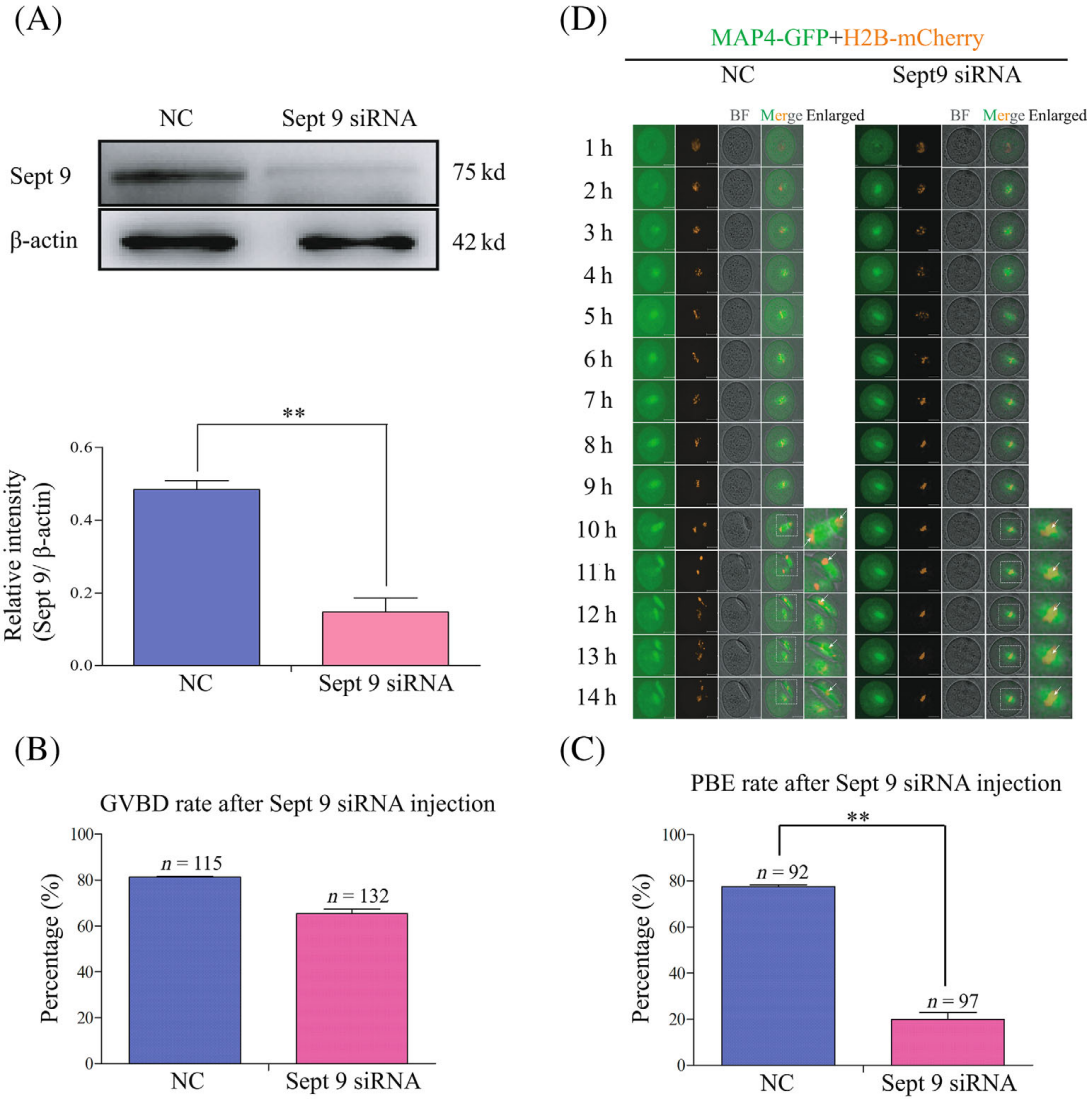
Depletion of Septin 9 affects the PBE rather than germinal vesicle breakdown (GVBD). (A) Western blotting results for Septin 9 and β‐actin in the *Sept 9* siRNA and control siRNA‐injected oocytes (150 oocytes per sample). The molecular weights of Septin 9 and β‐actin were about 75 and 42 Kd, respectively. The relative intensity of Septin 9 was determined by grayscale analysis using the software ImageJ. **p < 0.01. (B) Percentage of oocytes that underwent GVBD 2 h after following release from M2 with 200 μM IBMX in *Sept 9* siRNA (*n* = 115) and control siRNA (*n* = 132) injected oocytes. (C) Percentage of oocytes that underwent PBE 14 h after following release from M2 with 200 μM IBMX in *Sept 9* siRNA (*n* = 92) and control siRNA (*n* = 97) injected oocytes. **p < 0.01. (D) Representative time‐lapse confocal images of the MAP4‐GFP‐mRNA and H2B‐mcherry‐mRNA in *Sept 9* siRNA and control siRNA injected oocytes. The concentration of MAP4‐GFP‐mRNA used was 200 ng/μl and the concentration of H2B‐mcherry‐mRNA used was 50 ng/μl. Scale bars: 20 μm. Enlarged panels show high magnification views of the boxed areas. Arrows indicate the chromosome in oocytes. Scale bar: 1 μm (enlarged panels). Data are mean ± sem. All of the experiments were repeated at least three times, and representative results are shown.

To detect its function during oocyte meiotic maturation, Septin 9 was knocked down by microinjection of *Sept 9* siRNA. Compared with the control group, the protein level revealed by Western blotting (0.48 ± 0.02 vs. 0.15 ± 0.04) was significantly reduced in oocytes microinjected with *Sep 9* siRNA (**p < 0.01; Figure 2A). Compared with the control group (81.28 ± 0.30%), the *Sept 9* siRNA‐injected oocytes displayed a decreased GVBD rate (65.38 ± 1.95%), but there was no significant difference between these two groups (Figure 2B). However, the *Sept 9* siRNA‐injected oocytes displayed a reduced PBE rate. It was shown that 77.54 ± 0.87% of oocytes in the control group extruded the first polar body, while only 19.89 ± 3.19% of the *Sept 9* siRNA oocytes group completed maturation (**p < 0.01, Figure 2C). To trace the temporal and spatial changes of the first polar body extrusion (PBE), we next performed confocal live‐cell imaging for 14 h by labelling the fusion protein MAP4‐GFP and H2B‐mcherry. We found that the *Sept 9* siRNA‐injected oocytes did not extrude the first polar body, but the control siRNA‐injected oocytes extruded the first polar body at 10 h as normal (Figure 2D). These data suggested that Septin 9 may be necessary for the first PBE.

### 3.3 Septin 9 depletion impairs spindle dynamics, leading to MI arrest

**FIGURE 3:**
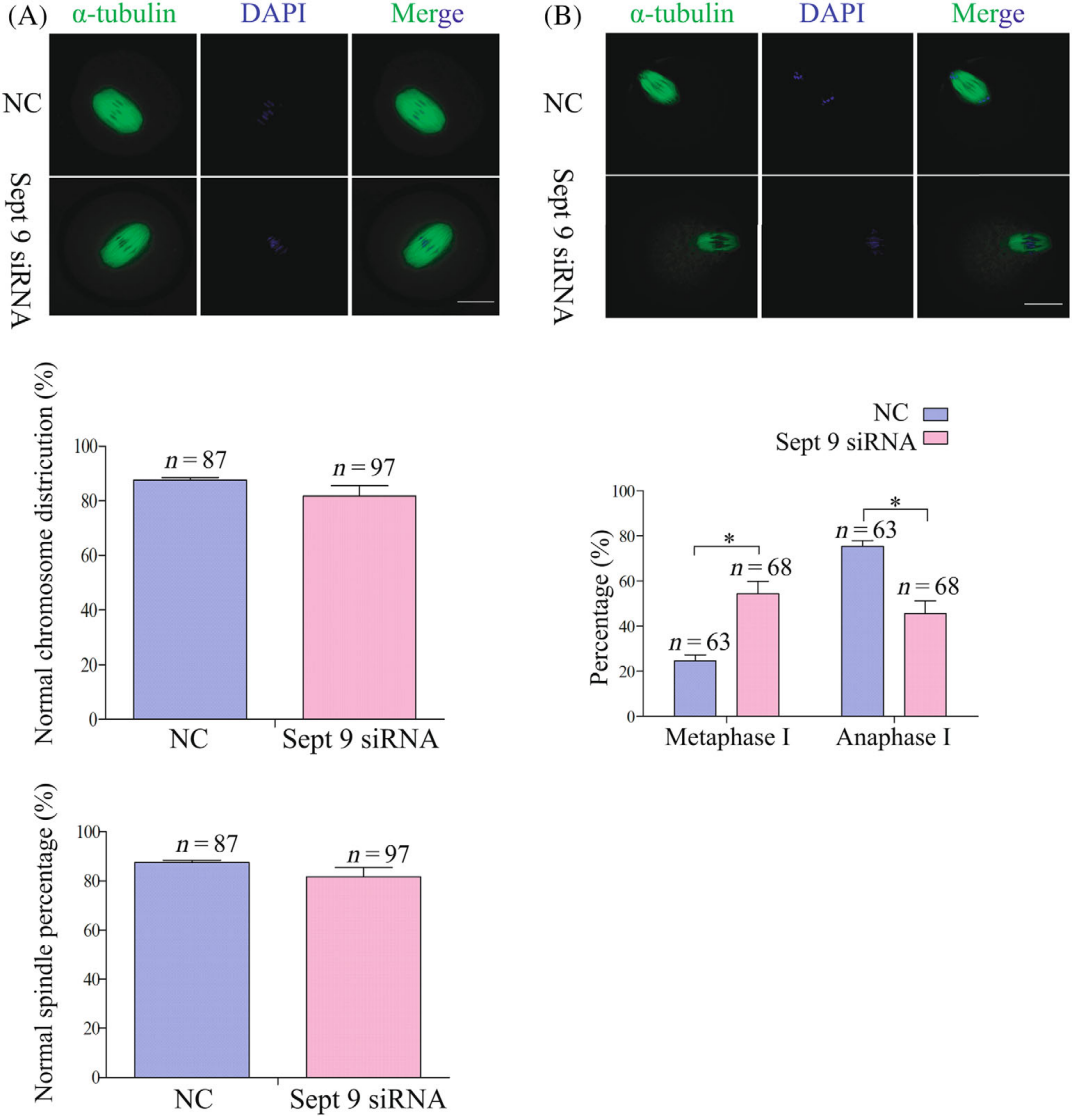
Septin 9 depletion does not impair spindle organization, but causes metaphase I arrest. (A) Confocal microscopy showing the spindle structures and distribution of chromosomes in *Sept 9* siRNA and control‐siRNA at 8 h, corresponding to the time points of metaphase I. Spindle and DNA were stained with α‐tubulin‐FITC antibody and DAPI, respectively. Scale bar: 20 μm. The percentages of normal chromosome distribution and spindle structures in the *Sept 9* siRNA group (*n* = 97) and control‐siRNA group (*n* = 87). (B) Confocal microscopy showing the spindle structures and distribution of chromosomes in *Sept 9* siRNA and control‐siRNA at 9.5 h, corresponding to the time points of anaphase I stages. Spindle and DNA were stained with α‐tubulin‐FITC antibody and DAPI, respectively. Scale bar: 20 μm. The percentage of metaphase I and anaphase I oocytes in *Sept 9* siRNA group (*n* = 68) and control‐siRNA group (*n* = 63). Data are mean ± sem. *p < 0.05. All of the experiments were repeated at least three times, and representative results are shown.

Because the percentage of PBE was reduced in Septin 9‐depleted oocytes, we next wanted to know the reason for this phenotype and the time when it happened. So, we next knocked down Septin 9 by injecting *Sept 9* siRNA and maintaining the oocytes in M2 medium with 200 μM IBMX for 24 h, while control‐siRNA injected oocytes were used as the control group. Then oocytes were released from IBMX, and the spindle structures and distribution of chromosomes in *Sept9* siRNA and control‐siRNA were observed. We collected *Sept 9* siRNA oocytes and control‐siRNA oocytes cultured for 8 h and 9.5 h, corresponding to the time points of MI and AI stages, to perform confocal microscopy. The results showed that there was no significant difference in the spindle structures and distribution of chromosomes between the *Sept 9* siRNA oocytes group and control‐siRNA oocytes group at 8 h (Figure 3A). However, we found that a large proportion of oocytes in the *Sept 9* siRNA group did not enter into the AI stage but were arrested at MI. The percentage of AI stage oocytes in the *Sept 9* siRNA group (*n* = 68, 45.62 ± 5.57%) was lower than that in the control‐siRNA group (*n* = 63, 75.33 ± 2.60%, *p < 0.05, Figure 3B). As a result, we suggested that the reduced PBE rate in Septin 9‐depleted oocytes was caused by failure of the MI‐AI transition, and that oocytes were arrested at MI.

### 3.4 Depletion of Septin 9 inhibits the MI/AI transition by regulating CCNB1 level

**FIGURE 4:**
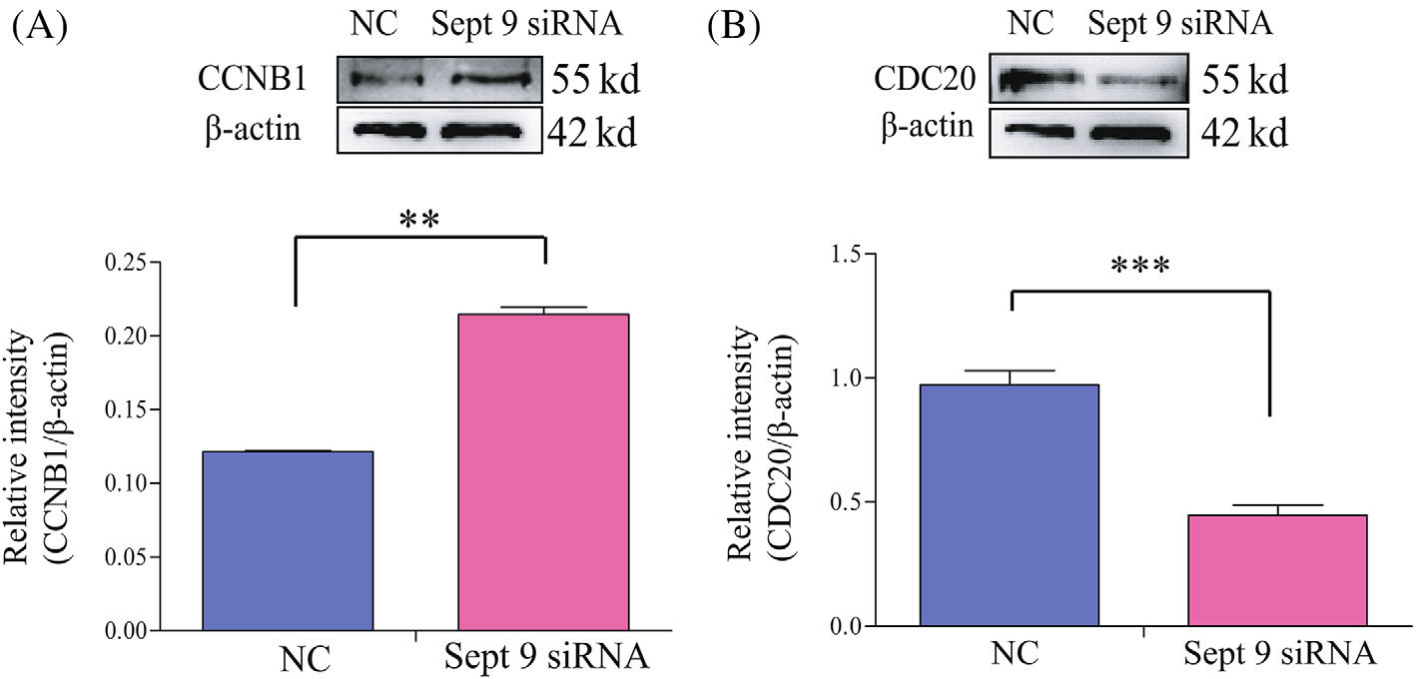
Depletion of Septin 9 inhibits MI/AI transition by regulating CCNB1 level. (A) Western blotting result for CCNB1 and β‐actin expression in Septin 9 siRNA and control‐siRNA injected oocytes at 9.5 h, corresponding to the time points of anaphase I stages (150 oocytes per sample). The molecular weights of CCNB1 and β‐actin were about 55 and 42 Kd, respectively. The relative intensity of CCNB1 was assessed by grayscale analysis using the software ImageJ. **p < 0.01. (B) Western blotting result for CDC20 and β‐actin expression in Septin 9 siRNA and control‐siRNA injected oocytes at 9.5 h, corresponding to the time points of anaphase I stages (150 oocytes per sample). The molecular weights of CDC20 and β‐actin were about 55 and 42 Kd, respectively. The relative intensity of CDC20 was assessed by grayscale analysis using the software ImageJ. ***p < 0.001. Data are mean ± sem. All of the experiments were repeated at least three times, and representative results are shown.

Considering that the expression of CCNB1 was maintained at a lower level during mouse oocyte AI, we designed experiments to analyse whether the failure of the MI/AI transition was caused by affecting the CCNB1 level in Septin 9‐depleted oocytes. We first knocked down Septin 9 by *Sept9* siRNA injection and cultured the oocytes in M2 medium with 200 μM IBMX for 24 h, and then oocytes were released from IBMX. Next, we collected *Sept 9* siRNA and control‐siRNA‐injected oocytes cultured for 9.5 h to perform Western blotting. The results showed that the expression of CCNB1 in *Sept 9* siRNA oocytes was significantly higher than that in the control‐siRNA group, and the relative expression levels of CCNB1 were 0.22 ± 0.02 and 0.12 ± 0.01, respectively, in the two groups (**p < 0.01, Figure 4A). And the expression of CDC20 in *Sept 9* siRNA oocytes was significantly lower than that in the control‐siRNA group, and the relative expression levels of CDC20 were 0.45 ± 0.04 and 0.97 ± 0.06, respectively, in the two groups (***p < 0.01, Figure 4B). Due to the fact that the degradation of CCNB1 is a prerequisite for the MI/AI transition, we speculated that depletion of Septin 9 may affect APC/C^CDC20^ and subsequently CCNB1 degradation. Therefore, the higher level of CCNB1 in *Sept 9* siRNA oocytes leads to the failure of AI entry at 9.5 h, thus oocytes were arrested at MI.

### 3.5 Depletion of Septin 9 results the abnormal kinetochore‐microtubule connection and SAC activation

**FIGURE 5:**
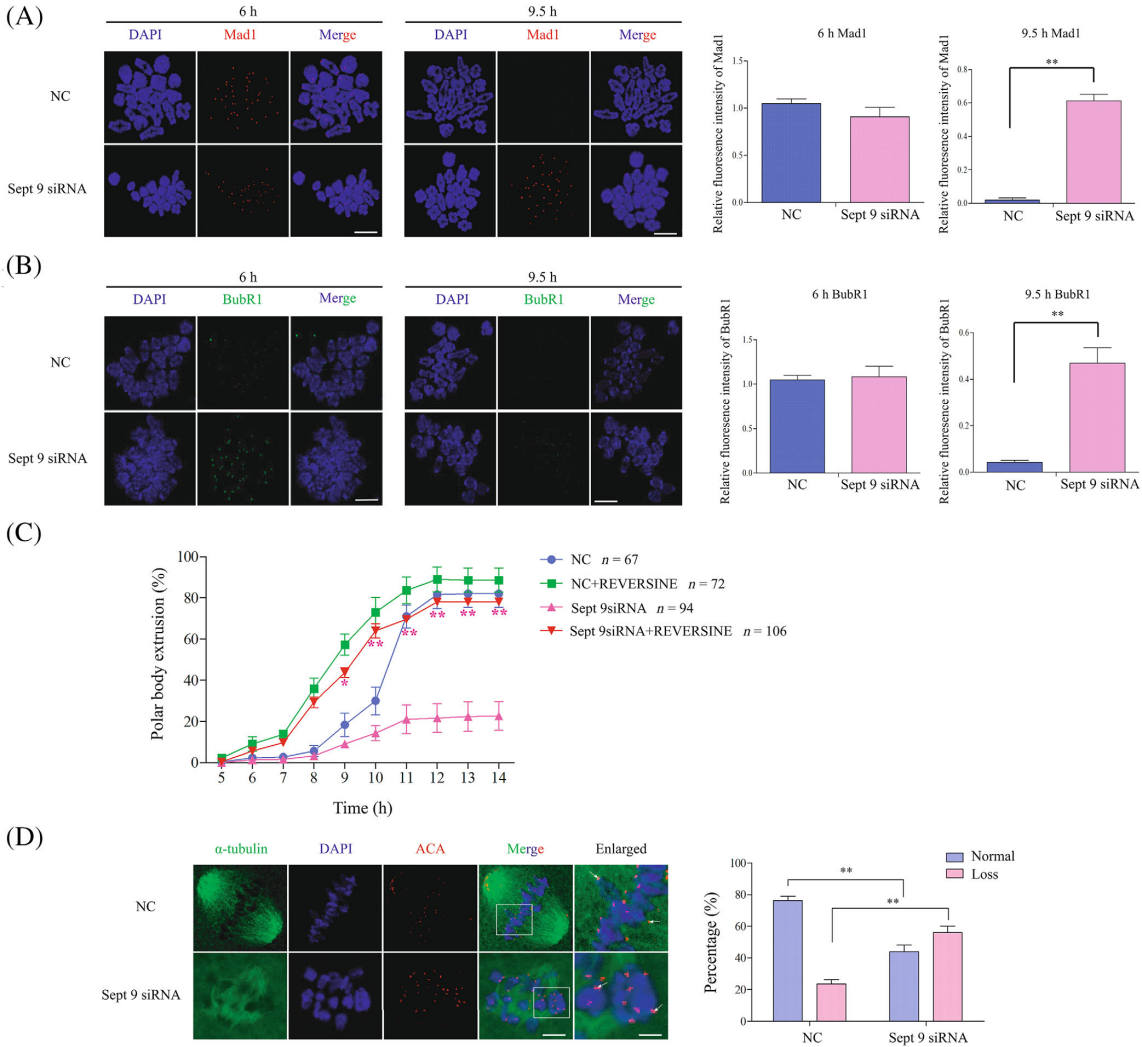
Depletion of Septin 9 results in abnormal connection between chromosome kinetochores and spindle microtubules and sustained spindle assembly checkpoint (SAC) activation. (A) Chromosome spread showing the activation of SAC‐Mad1 in *Sept 9* siRNA at 9.5 h. SAC‐Mad1 and DNA were stained with Mad1 antibody (Red) and DAPI (Blue), respectively. Scale bar: 5 μm. The bargraph represents the relative immunofluorescence intensity of Mad1 quantified in NC and *Sept 9* siRNA oocytes at 6 and 9.5 h following release from IBMX. (B) Chromosome spread showing the activation of SAC‐BubR1 in *Sept 9* siRNA at 9.5 h. SAC‐BubR1 and DNA were stained with BubR1 antibody (Green) and DAPI (Blue), respectively. Scale bar: 5 μm. The bargraph represents the relative immunofluorescence intensity of BubR1 quantified in NC and *Sept 9* siRNA oocytes at 6 and 9.5 h following release from IBMX. (C) The percentage of PBE of NC and Sept 9 siRNA oocytes after release from IBMX were incubated in M2 media containing 0.5 μM REVERSINE. (D) Confocal microscopy showing the spindle structures and distribution of chromosomes in Sept 9 siRNA at 8 h after cold treatment. Spindle and DNA were stained with α‐tubulin‐FITC antibody and DAPI, respectively. Scale bar: 5 μm. Enlarged panels show high magnification views of the boxed areas. Arrows indicate the kinetochores. Scale bar: 1 μm (enlarged panels). The bargraph represents the quantitative analysis of k‐MT attachments in NC and Sept 9 siRNA oocytes. ACA, anti‐centromeric antibodies. **p < 0.01. Data are mean ± sem. All of the experiments were repeated at least three times, and representative results are shown.

Generally, oocytes could finish the MI‐AI transition after stable kinetochore–microtubule connection and SAC inactivation.^42^, ^43^, ^44^ When SAC remains activated in oocytes, the activity of APC/C^CDC20^ is inhibited by SAC and then oocytes are arrested at MI.^45^ Activated APC/C^CDC20^ will degrade CCNB1 and thus oocytes finish the MI‐AI transition after SAC inactivation. Therefore, we designed experiments to detect the SAC activation in *Sept 9* siRNA oocytes at 6 and 9.5 h, respectively. As expected, we found that the signal of SAC proteins Mad1 and BubR1 at kinetochores were detected at 6 h in both *Sept 9* siRNA and control‐siRNA oocytes. By 9.5 h, the activation of SAC as indicated by localization of Mad1 and BubR1 on kinetochores was still detected in *Sept 9* siRNA oocytes but not in control oocytes (Figure 5A,B). Based on above results, we inhibited MPS1 to test whether the MI arrest could be rescued in Sept 9siRNA oocytes. The previous study has identified REVERSINE as potent inhibitors of MPS1, which plays an important role in SAC activity.^11^ After release from M2 medium containing IBMX inhibitor, we transferred the oocytes to M2 medium containing 0.5 μM of REVERSINE inhibitor for further cultivation. As expected, when adding REVERSINE to inhibit SAC activity, the progression of meiotic was accelerated. The first of PBE rate in Sept 9 siRNA + REVERSINE group (*n* = 106, 78.00 ± 0.58%) was significantly higher than that in Sept 9 siRNA group (*n* = 94, 22.67 ± 6.94%, **p < 0.01, Figure 5C). These results indicated that MI arrest caused by Sept 9 knockdown was caused by the continuous activation of SAC. We suggest that the continuous activation of SAC caused by depletion of Septin 9 inhibited the activity of APC/C^CDC20^, and thus CCNB1 was not degraded and then oocytes were arrested at MI. But, what is the possible reason for causing the continuous activation of SAC? Given that Septin 9 is a cytoskeleton protein, it can recruit other proteins to specific sites in the cytoplasm through the interaction between nucleotides, tubulin and actin. Thus Septin 9 can interfere with the function of other proteins. We speculated that the continuous activation of SAC might be caused by the abnormal connection between chromosome kinetochores and spindle microtubules in *Sept 9* siRNA oocytes.

We thus observed the stability of connections between kinetochores and spindle microtubules in *Sept 9* siRNA oocytes and control siRNA oocytes which were cultured for 8 h under cold treatment at 4°C. As shown in Figure 5D, confocal microscopy showed that the kinetochore‐microtubule polymerization in *Sept 9 siRNA* oocytes was impaired after cold treatment. We found that the ACA was distributed widely at the equatorial plate at the MI stage in *Sept 9* siRNA oocytes, and most of the kinetochores did not connect to the bilateral microtubules. Besides, compared to the control siRNA group, the kinetochores that were devoid of microtubules were observed in *Sept 9* siRNA oocytes. These results proved that depletion of Septin 9 damaged the stability of kinetochore‐microtubule connections, leading to continuous activation of SAC and inhibition of CCNB1 degradation, and then oocytes became arrested at the MI stage.

### 3.6 Exogenous *GFP‐Sept* 9 mRNA injection can partly rescue the MI arrest caused by *Sept 9* siRNA

We wanted to know whether exogenous Septin9‐GFP expression could rescue the MI arrest caused by *Sept 9* siRNA. Firstly, we analysed the effect of *Sept 9* mRNA injection on the PBE. The results showed that there was no significant difference in spindle structure and chromosome distribution between the *Sept 9‐GFP* mRNA group and control siRNA group. The percentages of GVBDs and PBEs in the *Sept 9‐GFP* mRNA group were similar to those in the control siRNA group (Figure S1A). Then, we collected oocytes which were cultured for 9.5 h to analyse the expression of CDC20 and CCNB1 by Western blot in the two groups and we found that there was no significant difference in the expression of CDC20 and CCNB1 (Figure S1B).

**FIGURE 6:**
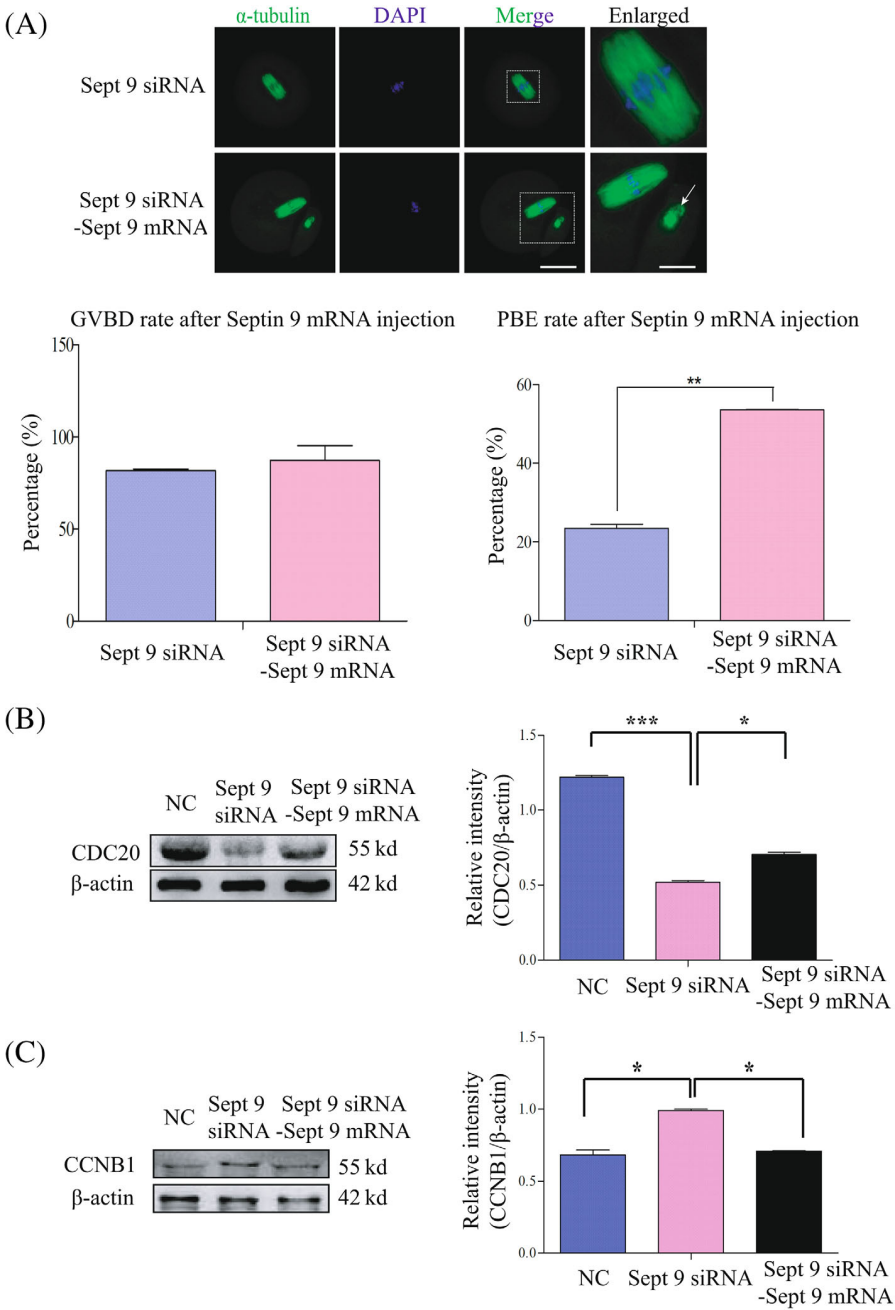
Exogenous *GFP‐Sept* 9 mRNA injection can partly rescue the MI arrest caused by *Sept 9* siRNA. (A) Confocal microscopy showing the spindle structures and distribution of chromosomes in mixes of *Sept 9* siRNA and *GFP‐Sept 9* mRNA and *Sept 9* siRNA injected oocytes at 14 h. Spindle and DNA were stained with α‐tubulin‐FITC antibody and DAPI, respectively. Scale bar: 20 μm. Enlarged panels show high magnification views of the boxed areas. Arrows indicate the kinetochores. Scale bar: 1 μm (enlarged panels). The percentages of germinal vesicle breakdown and PBE are shown in both groups. (B) Western blotting result for CDC20 and β‐actin expression in NC oocytes, mix of *Sept 9* siRNA and *GFP‐Sept 9* mRNA and *Sept 9* siRNA injected oocytes (150 oocytes per sample). The molecular weights of CDC20 and β‐actin were about 55 and 42 Kd, respectively. The relative intensities of CDC20 were determined by grayscale analysis using the software ImageJ. Data are mean ± sem. *p < 0.05; ***p < 0.001. (C) Western blotting result for CCNB1 and β‐actin expression in NC oocytes, mix of *Sept 9* siRNA and *GFP‐ Sept 9* mRNA and *Sept 9* siRNA injected oocytes (150 oocytes per sample). The molecular weights of CCNB1 and β‐actin were about 55 and 42 Kd, respectively. The relative intensities of CCNB1 were determined by grayscale analysis using the software ImageJ. Data are mean ± sem. *p < 0.05. All of the experiments were repeated at least three times, and representative results are shown.

Based on the above results, we microinjected *GFP‐Sept 9 mRNA* into *Sept 9* siRNA oocytes which were cultured in medium containing GVBD inhibitor for 24 h, and then we continued to culture oocytes in medium containing the inhibitor for 4 h. At last, we released oocytes into the M2 medium without inhibitor and cultured oocytes for 14 h. As we expected, most of oocytes finished the MI/AI transition and extruded the first polar body in the mix of the *Sept 9* siRNA and *Sept 9‐GFP* mRNA‐injection group (Figure 6A). Meanwhile, the results of Western blotting showed that the expression of CDC20 was rescued and the expression of CCNB1 was reduced after exogenous *GFP‐Septin 9 mRNA* injection. The relative intensities of CDC20 in NC group, *Sept 9* siRNA and *Sept 9* siRNA + *Sept 9‐GFP* mRNA groups were 1.22 ± 0.01, 0.52 ± 0.01 and 0.71 ± 0.02, respectively. The relative intensities of CCNB1 in NC group, *Sept 9* siRNA group and *Sept 9* siRNA + *Sept 9*‐GFP mRNA group were 0.68 ± 0.04, 0.99 ± 0.01 and 0.71 ± 0.01 (*p < 0.05, ***p < 0.001, Figure 6B,C). The above results suggested that the effect of endogenous Septin 9 knockdown on the PBE rate was partly rescued by the exogenous *Sept 9*‐GFP mRNA injection.

### 3.7 MI arrest caused by Septin 9 depletion can be partly rescued by Myc‐*Cdc20* mRNA injection

**FIGURE 7:**
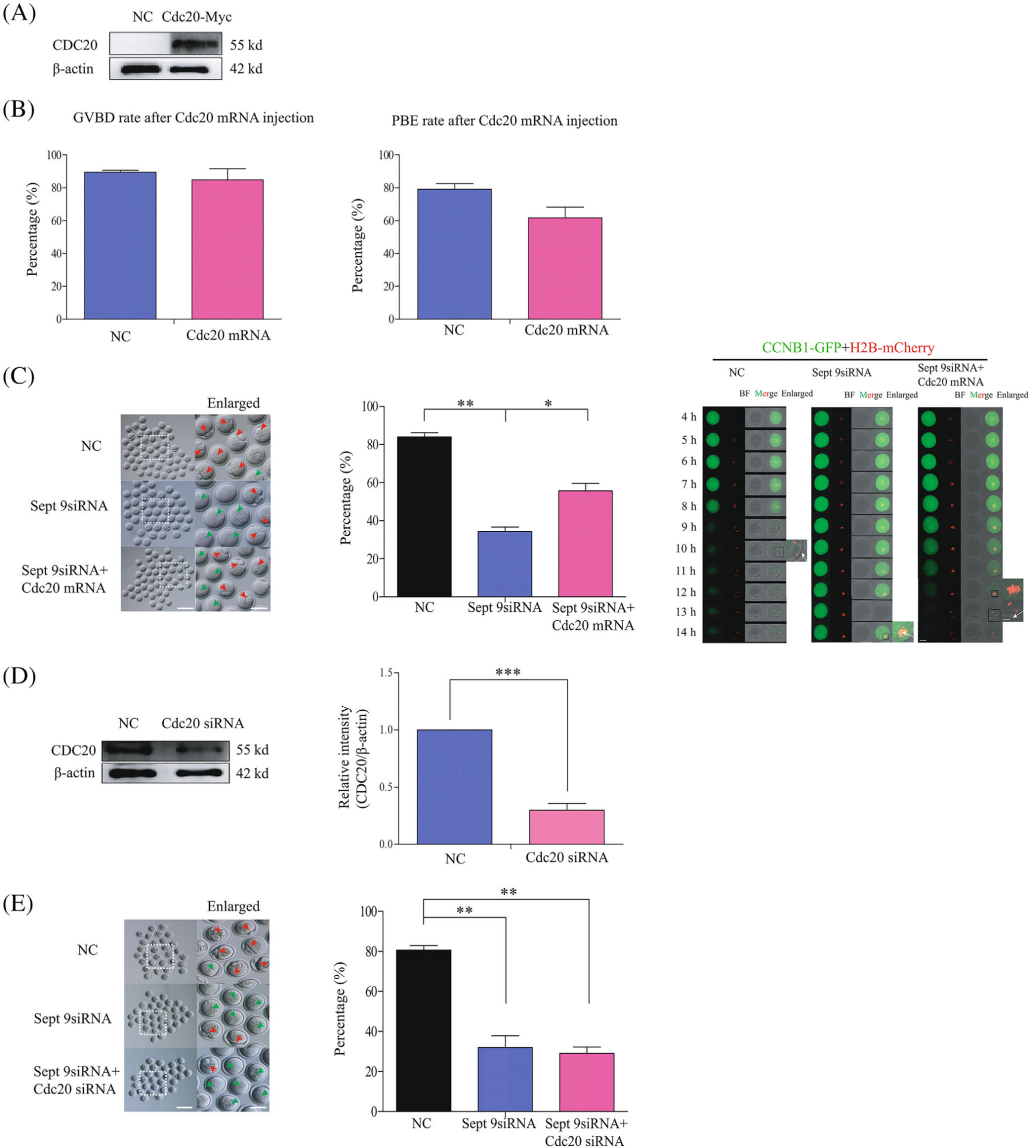
Metaphase I arrest caused by Septin 9 depletion can be partly rescued by *Myc‐Cdc20* mRNA injection. (A) Western blotting of CDC20 and β‐actin in the *Myc‐Cdc20* mRNA and control mRNA‐injected oocytes (150 oocytes per sample). The molecular weights of CDC20 and β‐actin were about 55 and 42 Kd, respectively. (B) The percentages of oocytes undergoing germinal vesicle breakdown and PBE in *Myc‐Cdc20* mRNA and control mRNA injected oocytes after release from IBMX. (C) Morphology of PBE oocytes from the *Sept 9* siRNA group, mixes of *Sept 9* siRNA and *Myc‐Cdc20* mRNA group and control group 14 h after release from IBMX. Enlarged panels show high magnification views of the boxed areas. Scale bar: 1 μm (enlarged panels). Rea arrows indicate the first polar body extrusion, while green arrows indicate the first polar body was not extruded. The percentages of PBE are shown in three groups. Scale bars: 100 μm. Data are mean ± sem. *p < 0.05; **p < 0.01. Representative time‐lapse confocal images of the CCNB1‐GFP‐mRNA and H2B‐mcherry‐mRNA in *Sept 9* siRNA, mixes of *Sept 9* siRNA and *Myc‐Cdc20* mRNA and control siRNA injected oocytes. The concentration of CCNB1‐GFP‐mRNA used was 40 ng/μl and the concentration of H2B‐mcherry‐mRNA used was 50 ng/μl. Scale bars: 20 μm. Enlarged panels show high magnification views of the boxed areas. Arrows indicate the chromosome in oocytes. Scale bar: 1 μm (enlarged panels). (D) Western blotting results for CDC20 and β‐actin in the *Ccd20* siRNA and control siRNA injected oocytes (150 oocytes per sample). The molecular weights of CDC20 and β‐actin were about 55 and 42 Kd, respectively. The relative intensity of CDC20 was determined by grayscale analysis using the software ImageJ. ***p < 0.001. (E) Morphology of PBE oocytes from the *Sept 9* siRNA group, mix of *Sept 9* siRNA and *Cdc20* siRNA group and control group 14 h after release from IBMX. Enlarged panels show high magnification views of the boxed areas. Scale bar: 1 μm (enlarged panels). Rea arrows indicate the first polar body extrusion, while green arrows indicate the first polar body was not extruded. The percentages of PBE were shown in three groups. Scale bars: 100 μm. Data are mean ± sem. **p < 0.01. All of the experiments were repeated at least three times, and representative results are shown.

To confirm whether the MI arrest caused by Septin 9 depletion could be rescued by *Myc‐Cdc20* mRNA injection, we first constructed the *Myc‐Ccd20* plasmid and proved that the exogenous *Myc‐Cdc20* mRNA could be successfully expressed in oocytes (Figure 7A). And then we observed and analysed the percentage of GVBDs and PBEs in *Myc‐Cdc20* mRNA‐injected oocytes and control mRNA‐injected oocytes. The results showed that there was no significant difference in the GVBD rates (84.78 ± 6.73% vs. 89.58 ± 1.12%) and PBE rates (61.73 ± 6.44% vs. 79.21 ± 3.30%) between two groups (Figure 7B). Therefore, we designed experiments to analyse the percentages of PBE in *Sept 9* siRNA‐injected oocytes, mix of *Sept 9* siRNA and *Myc‐Cdc20* mRNA‐injected oocytes and control siRNA‐injected oocytes. The results showed that the percentage of PBE in the *Sept 9* siRNA oocytes was lower than that in the other two groups, and the percentage of PBE in control siRNA‐injected oocytes was still higher than that in the mix of *Sept 9* siRNA and *Myc‐Cdc20* mRNA‐injected oocytes. The percentages of PBE rates in the three groups were 34.31 ± 2.23%, 55.70 ± 3.85% and 83.97 ± 2.06%, respectively. To trace the temporal and spatial changes of the first PBE, we next performed live‐cell confocal imaging for 14 h by labelling the fusion protein CCNB1‐GFP and H2B‐mcherry. We found that the *Sept 9* siRNA‐injected oocytes did not extrude the first polar body, but the control siRNA‐injected oocytes and the mix of *Sept 9* siRNA and *Myc‐Cdc20* mRNA‐injected oocytes extruded the first polar body (Figure 7C).

Next, to confirm that the lower percentage of PBE caused by Septin 9 depletion could be result from the lower expression of CDC20, we determined the CDC20 knockdown efficiency. The results showed that the expression of CDC20 was significantly reduced in oocytes microinjected with *Cdc20* siRNA. The relative intensities of CDC20 in the *Cdc20* siRNA‐injected group and control group were 0.30 ± 0.06 and 0.99 ± 0.01, respectively (***p < 0.001, Figure 7D). And then we again analysed the percentages of PBE in *Sept 9* siRNA‐injected oocytes, mix of *Sept 9* siRNA and *Cdc20* siRNA‐injected oocytes and control siRNA‐injected oocytes. The results showed that the percentage of PBE in the mix of *Sept 9* siRNA and *Cdc20* siRNA‐injected oocytes was lower than that in the other two groups. The percentages of PBE in three groups were 31.98 ± 5.92%, 29.03 ± 3.22% and 80.64 ± 2.35%, respectively (Figure 7E). These results further revealed that the failure of the MI/AI transition caused by Septin 9 depletion and CCNB1 accumulation might be related to the decrease of CDC20 expression levels.

## 4 DISCUSSION

When *Sept 9* gene expression is abnormal or absent, cell division can be severely affected.^46^ Studies have confirmed that knockout of the *Sept 9* gene affects cytoplasmic division, spindle assembly and the production of polyploidy or aneuploidy cells, which will interfere with the cell's genomic stability.^30^, ^31^, ^32^ In this study, we reveal an important role of Septin 9 in the regulation of the MI‐AI transition, a critical step in mouse oocyte maturation. Septin 9 was expressed at a gradually rising level from the GV to MII stages and it was observed to distribute in the cytoplasm and spindle (Figure 1C). This result is consistent with the previously reported co‐localization of Septin 9 filaments and actin filaments.^41^ These results lead us to consider that Septin 9 might play an important role in mouse oocyte meiotic maturation. Therefore, Septin 9 was knocked down by microinjection of *Sept 9* siRNA to explore its function during mouse oocyte meiotic maturation. Compared with control siRNA‐injected oocytes, we found that a large proportion of Septin 9 siRNA‐injected oocytes did not extrude the first polar body (Figure 2D).

Spindle assembly and chromosome separation regulation are the most important events during mouse oocyte meiotic maturation. Chromosome segregation errors through non‐disjunction and disorganized assembly of the spindle will result in aneuploid oocytes and early pregnancy loss.^47^, ^48^ Therefore, we focused on studying spindle assembly and chromosome separation to explain the reduction of the first PBE in *Sept 9* siRNA oocytes. To our surprise, the result showed that there was no difference in spindle assembly between control and Sept 9 knockdown oocytes at MI stage (Figure 3A), but the chromosome separation failed and the cytoplasm did not divide in Sept9 siRNA group at 9.5 h. Meanwhile, we found that the reduced PBE in *Sept 9*‐depleted oocytes was caused by failure of the MI‐AI transition, which is regulated by the APC/C^CDC20^ (Figure 3B). Therefore, we suggest that Septin 9, despite localized on spindle, is not essential for spindle assembly, but it is involved in spindle microtubule pulling of chromosomes towards the poles. APC/C can promote MI‐AI cell cycle transition.^8^ Besides that, APC/C has ubiquitin activity only when it binds to CDC20 or CDH1.^22^ When the kinetochores on chromosomes are properly connected to the spindle microtubules at MI, the ubiquitase activity of APC/C^CDC20^ will lead to the degradation of CCNB1 and securin and promote metaphase‐to‐anaphase transition. Considering the fact that the *Sept 9* siRNA knockdown group had a lower PBE rate and the degradation of CCNB1 is the prerequisite for extrusion of the first polar body, we speculated that CCNB1 might not be degraded in the *Sept 9* siRNA group in MI arrested oocytes. As expected, we showed that CCNB1 failed to degrade in Septin 9‐depleted oocytes at 9.5 h (Figure 4). It is well known that APC/C^CDC20^ is the dominant activator at the metaphase‐to‐anaphase transition in meiosis, which promotes the degradation of CCNB1.^49^ So, we next focused on the activity of APC/C^CDC20^ and speculated that the activity of APC/C^CDC20^ may be inhibited so that CCNB1 could not be degraded on time. Some studies had shown that CDC20 is the downstream protein of SAC.^21^ SAC is a highly conserved supervision mechanism, that can delay the onset of anaphase until all sister chromosomes are properly aligned on the equatorial plates in both mitosis and meiosis.^7^, ^8^, ^9^ We, thus, analysed the activation of SAC in *Sept 9* siRNA at 6 and 9.5 h (Figure 5A–C). The results showed that the continuous activation of SAC caused by depletion of Septin 9 inhibited the activity of APC/C^CDC20^, therefore CCNB1 could not be degraded on time and then oocytes were arrested at MI. The unstable connection between kinetochores and microtubules could lead to continuous activation of SAC. The present study showed unstable connections between kinetochores and microtubules by cold treatment and thus continuous activation of SAC to inhibit the MI‐AI transition in *Sept 9* knockdown oocytes arrested at MI. In addition, we also found that exogenous *Sept 9*‐GFP mRNA and *MyC‐Cdc20* mRNA injection could partly rescue the failure of the MI‐AI transition caused by *Sept 9* siRNA. Besides, the expression levels of CDC20 and CCNB1 were also rescued. These results suggest that MI arrest and CCNB1 non‐degradation phenotypes caused by *Sept9* siRNA may be related to CDC20 regulation.

**FIGURE 8:**
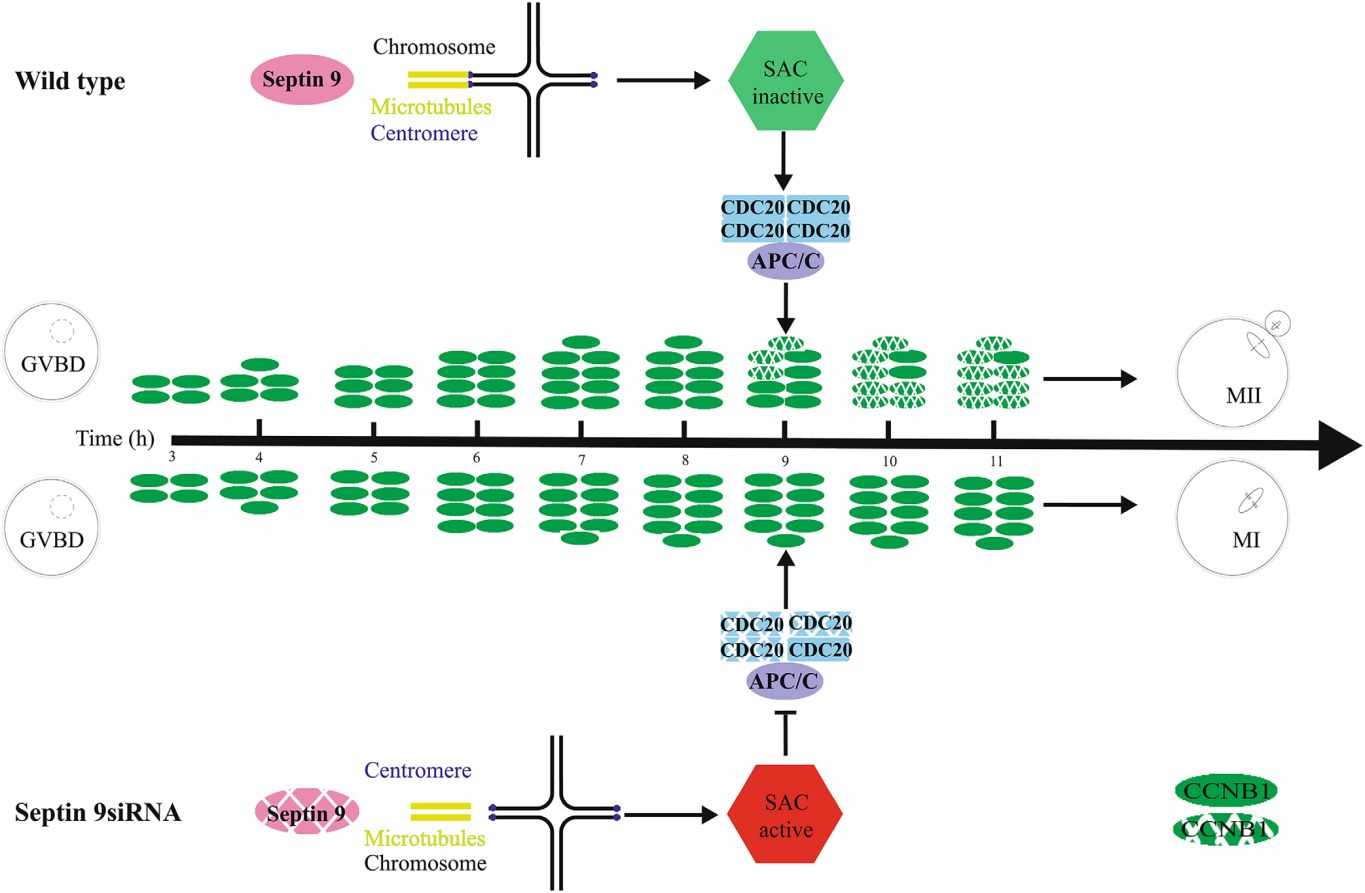
The mechanism of Septin 9 depletion‐caused MI/AI transition failure during meiotic maturation in mouse oocytes. Septin 9 may play an important role in regulating the MI/AI transition by influencing the stability of kinetochore‐microtubule connections in mouse oocytes. In wild types, Septin 9 allows CCNB1 degradation, which in turn causes metaphase I‐to‐anaphase I transition and the first polar body extrusion. Conversely, depletion of Septin 9 disrupts CCNB1 degradation by sustaining SAC activation and downregulating APC/C^CDC20^ activity. Sustained SAC activation is caused by unstable connections between kinetochores and microtubules in Septin 9‐depleted oocytes. Accordingly, Septin 9‐depleted oocytes arrested at metaphase I stage and did not extrude the first polar body.

In conclusion, Septin 9 depletion may disrupt proper kinetochore‐microtubule connections, and cause meiotic oocyte maturation failure. This phenotype may be caused by sustained SAC activation, subsequent failure of APC/C^CDC20^ activation and impeded CCNB1 degradation, which finally impairs MI‐AI transition in meiotic oocytes (Figure 8). The molecular mechanisms of how Septin 9 regulates the connection between kinetochores and spindle microtubules needs further clarification.

## AUTHOR CONTRIBUTIONS

Li Chen performed the major experiments, analysed the data and wrote the manuscript; Ying‐Chun Ouyang, Jia‐Ni Guo, Lin‐Jian Gu, Zhi‐Ming Han, Zhen‐Bo Wang and Yi Hou contributed to technical assistance; Heide Schatten revised the manuscript. Qing‐Yuan Sun designed and organized the study and revised the manuscript. All authors read and approved the final manuscript.

## FUNDING INFORMATION

This study was supported by National Natural Science Foundation of China (32570854) and Natural Science Foundation of Shandong Province (ZR2021ZD33).

## CONFLICT OF INTEREST

The authors declare that the research was conducted in the absence of any commercial or financial relationships that could be construed as a potential conflict of interest.

## Supporting information


**FIGURE S1** The effect of Sept 9 mRNA injection on the PBE. (A) Confocal microscopy showing the spindle structures and distribution of chromosomes in *GFP‐ Sept 9* mRNA and control mRNA injected oocytes at 14 h. Spindle and DNA were stained with α‐tubulin‐FITC antibody and DAPI, respectively. Scale bar: 20 μm. The percentages of GVBD and PBE were shown in both groups. (B) Western blotting result for CDC20, CCNB1 and β‐actin expression in the *Sept 9‐GFP* mRNA and control‐mRNA injected oocytes (150 oocytes per sample). The molecular weights of CDC20, CCNB1 and β‐actin were about 55, 55 and 42 Kd, respectively. The relative intensities of CDC20 and CCNB1 were determined by grayscale analysis using the software ImageJ. Data are mean ± sem. All of the experiments were repeated at least three times, and representative results are shown.

## Data Availability

The data used to support the findings of this study are available from the corresponding author upon request.
