# Supplementary figures and images for: Septin 9 controls CCNB1 stabilization via APC/CCDC20 during meiotic metaphase I/anaphase I transition in mouse oocytes
Source: Cell Prolif. 2022 Nov 10;56(2):e13359. doi: 10.1111/cpr.13359 (PMC9890537; doi:10.1111/cpr.13359)

A

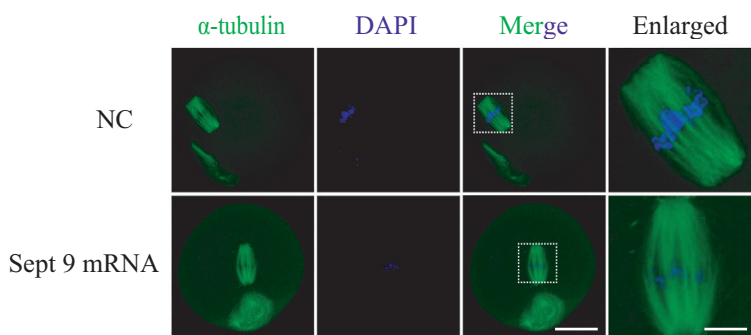

GVBD rate after Septin 9 mRNA injection

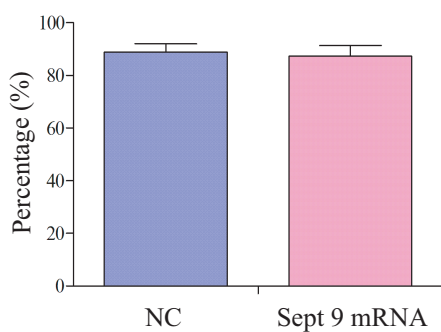

PBE rate after Septin 9 mRNA injection

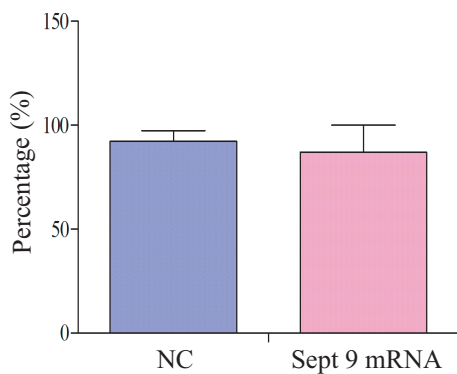

B

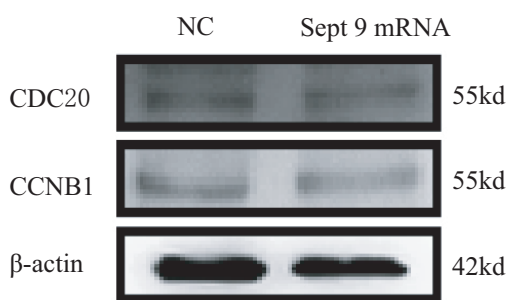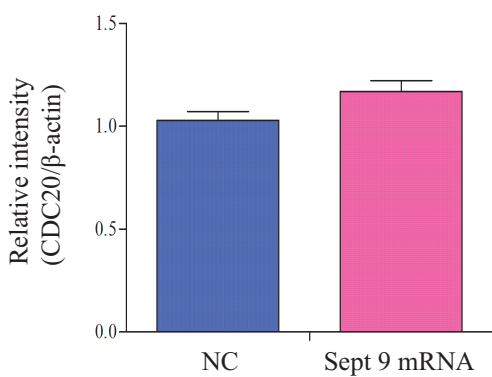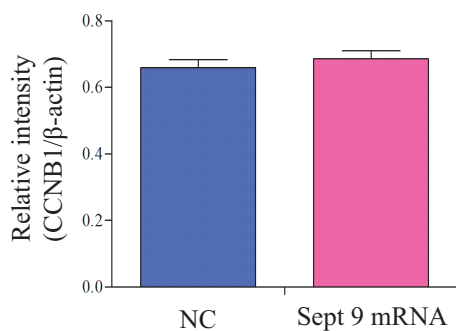

Supplement: Supplementary file 1 — FIGURE S1 The effect of Sept 9 mRNA injection on the PBE. (A) Confocal microscopy showing the spindle structures and distribution of chromosomes in GFP‐ Sept 9 mRNA and control mRNA injected oocytes at 14 h. Spindle and DNA were stained with α‐tubulin‐FITC antibody and DAPI, respectively. Scale bar: 20 μm. The percentages of GVBD and PBE were shown in both groups. (B) Western blotting result for CDC20, CCNB1 and β‐actin expression in the Sept 9‐GFP mRNA and control‐mRNA injected oocytes (150 oocytes per sample). The molecular weights of CDC20, CCNB1 and β‐actin were about 55, 55 and 42 Kd, respectively. The relative intensities of CDC20 and CCNB1 were determined by grayscale analysis using the software ImageJ. Data are mean ± sem. All of the experiments were repeated at least three times, and representative results are shown. [file CPR-56-e13359-s001.pdf]
